# Neurointerventional infusion of hemoglobin oxygen carrier prevents brain damage from acute cerebral ischemia in rats

**DOI:** 10.3389/fsurg.2023.1050935

**Published:** 2023-03-03

**Authors:** Hong Gao, Han Peng, Hua Yang, Qiuping Li, Xin Xiang

**Affiliations:** ^1^ Department of Neurosurgery, Zhongshan Hospital (Xiamen), Fudan University, Xiamen, China; ^2^ Department of Neurosurgery, Affiliated Hospital of Guizhou Medical University, Guiyang, China

**Keywords:** hemoglobin oxygen carrier (HBOC), neurointervention, microcatheter perfusion, acute cerebral ischemia, rats

## Abstract

**Aim:**

To save brain cells in acute cerebral infarction by injecting hemoglobin oxygen carrier (HBOC) into the blood vessel blockage of the cerebral infarction site through a microcatheter.

**Methods:**

120 male rats were divided into four groups: control (CTRL), ischemia (I), ischemia + low perfusion (I + LP), and ischemia + high perfusion (I + HP). Perfusion groups (ischemia, I + LP, and I + HP) underwent MCAO surgery with intraluminal monofilament. These groups were subdivided into 6 h, 12 h, and 24 h (*n* = 10/group). RT-PCR, Western-Blot, immunohistochemistry, and apoptosis assays were used to detect apoptosis, hypoxia range and extent, and ischemia.

**Results:**

Compared with the I group, the neurological deficit sign scores of the I + HP group were statistically significant at 12 h. Compared with the I group, the neurological deficit sign scores of the I + LP group and the I + HP group were statistically significant at 24 h. At all time points, compared with the I group and the I + LP group, Caspase-3, HIF-1α, and Cytochrome C protein levels were significantly decreased in the I + HP group. Bcl-2 and BAX mRNA levels were also significantly decreased in the same group. TNF-α, IL-6, and IL-1β cytokines were significantly decreased in the I + HP group as well. The infarct size of rats in the I + HP group was smaller than that of the I + LP group, which was smaller than ischemia alone. Time of perfusion had an obvious effect as infarct size was smaller with longer perfusion. The number of Nissl stained cells in the I + HP group was increased compared with the ischemia and the I + LP group, and was proportional to the time of perfusion.

**Conclusion:**

Time- and rate-controlled perfusion of HBOC to acutely occluded cerebral vascular regions through microcatheters can effectively protect ischemic brain tissue in rats.

## Introduction

Although many new technologies have accelerated revascularization in acute cerebral infarctions, the process from acute cerebral infarction to reperfusion is often delayed due to various factors (1). Vascular reconstruction is an event correlated with ischemia and reperfusion. Whether thrombolysis or thrombus removal, vascular reconstruction takes time and may fail. Therefore, it is necessary to preserve the brain before revascularization occurs. At present, there is no research that looks at infusing HBOC into acute cerebral infarctions as this technique has not yet manifested itself in the clinic. Microcatheter technology allows the possibility of delivering HOBC to a cerebral infarction, which may prove beneficial.

Due to the increasing maturity of neuro-interventional technology, catheters can be used to transport blood flow to ischemic brain tissue across occluded blood vessels (2, 3). Hemoglobin oxygen carrier (HBOC) has the ability to carry oxygen and release oxygen, whose volume is much smaller than that of normal red blood cells and viscosity is low. It is easy to perfuse effectively through tiny blood vessels that ordinary blood cells cannot pass through (4, 5). HBOC can be used to supply oxygen to the microvasculature of the brain, allowing ischemic brain tissue to be protected until recanalization (6).

This study used microcatheter technology, without injecting thrombolytic or anticoagulant drugs, to inject HBOC into the blood vessel blockage of cerebral infarctions of rats in order to investigate whether this way can effectively alleviate the symptoms of cerebral ischemia, thereby reducing the death of cerebral nerve cells caused by subsequent hypoxia.

## Methods

All experiments were approved by the Animal Care Welfare Committe of the Guizhou Medical University (No: 1600286).

### Grouping

Eight week old male Sprague–Dawley (SD) rats (about 250 g), that were similar in age, size, and body weight were randomly divided into 12 groups. The groups included control (CTRL), ischemia (I), ischemia + low perfusion (I + LP), and ischemia + high perfusion (I + HP). These groups were then subdivided into 6 h, 12 h, 24 h of perfusion groups (*n* = 10/group, 120 total).

### Ischemia surgery

**Figure 1:**
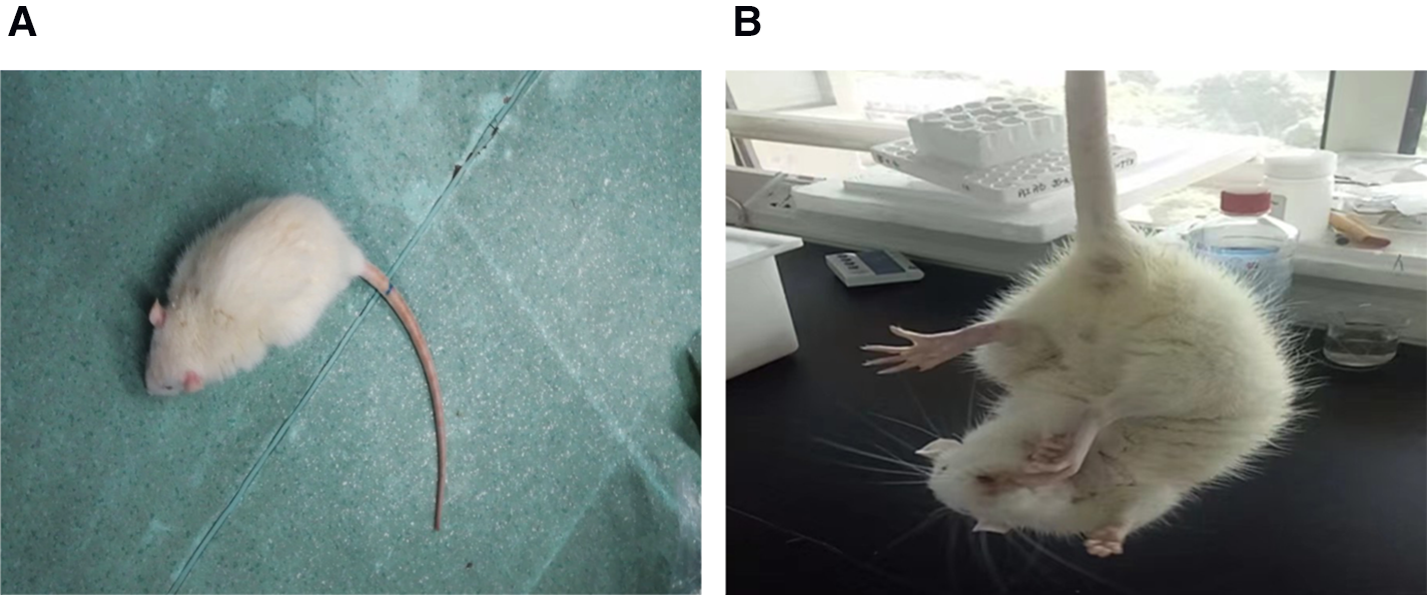
In the I group, when the rat was placed on the ground, they crawled and circled around the contralateral side of the damage. When the rat was lifted off the ground by the tail, the contralateral forelimbs were internally rotated and adducted. These behaviors are different from those in the control group.

Perfusion groups (ischemia, I + LP, and I + HP) underwent MCAO surgery with intraluminal monofilament. Inhalation anesthesia was induced in rats with 5% isoflurane (JW Pharm, Seoul, South Korea) and maintained with 3% isoflurane. A fishing line (MSRC35B200PK50, RWD) was inserted into the right internal carotid artery of each rat. Once through, a knot was gently tied at the distal end of the common carotid artery. The tethered thread was gently pushed by ophthalmic forceps to a calculated distance from the bifurcation of the blood vessel. When the insertion depth was 18 mm, the thin line of the distal end of the common carotid artery was tightened. The rats were anesthetized and subjected to a sham surgery in the control group. The rats were observed and tissues were collected 24 h postoperatively. At the end of treatment, neurological deficit sign scores were obtained (Figure 1, Table 1) (7, 8). Afterwards, animals were sacrificed and the vascular line was removed. Pathological light microscopy was used to observe changes in brain tissue sections. Rats were sacrificed 24 h postoperatively and underwent euthanasia *via* a calculated chamber displacement rate of 30% of the chamber volume per minute using 100% CO_2_.

**Table 1:** Neurological deficit sign score*.

Score	Symptom
0	The rats had no symptoms of nerve damage.
1	The rats could not fully extend the contralateral front paw.
2	The rats turned to the opposite side in a circle.
3	The rats dumped to the opposite side.
4	The rats could not walk spontaneously, loss of consciousness.

* According to Longa and Bederson's 5-point system.

### Preparation of HBOC

**Figure 2:**
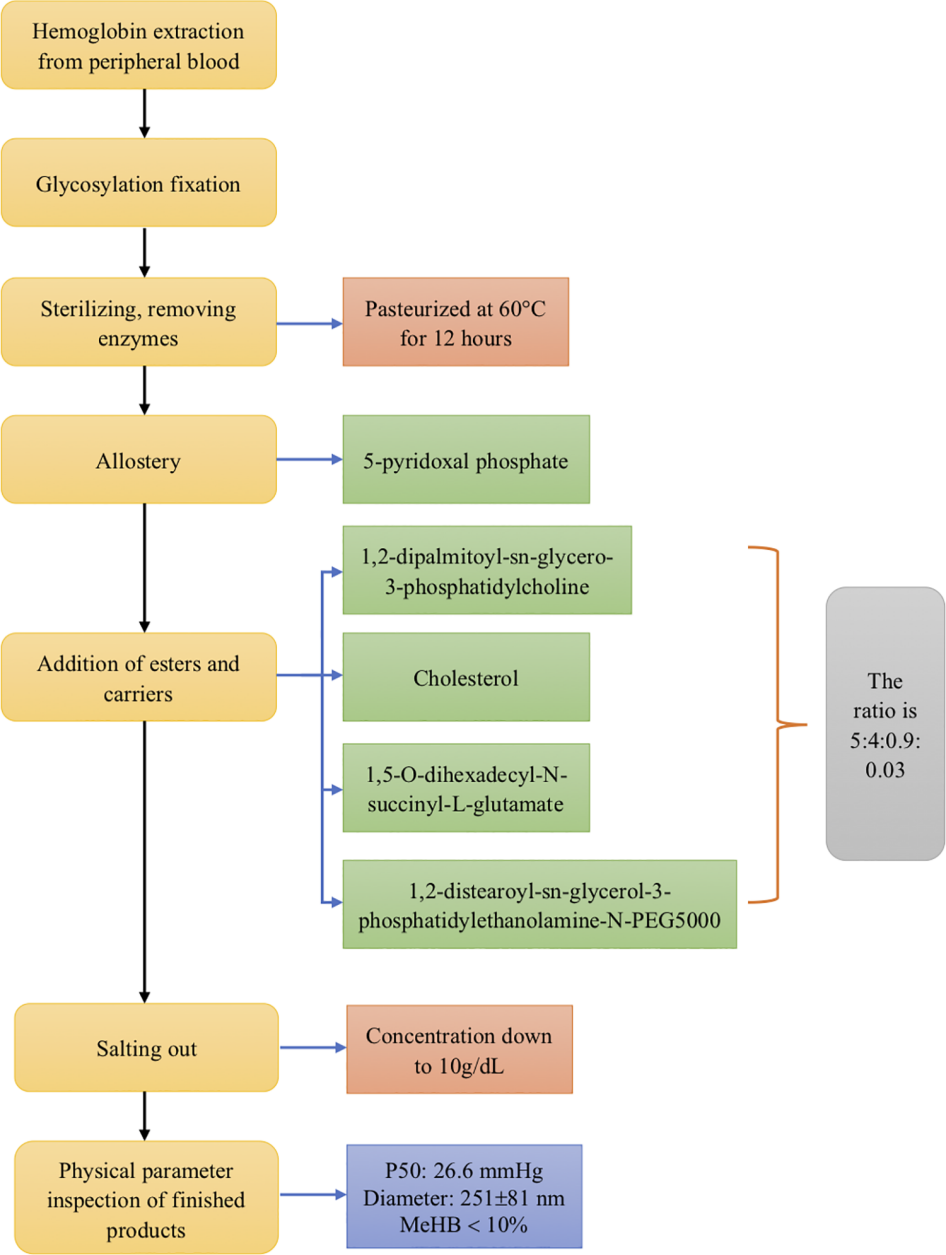
The flow chart of HBOC preparation.

Hemoglobin human (H7379-10G, sigma) was extracted by 0.0200 g into a 5 ml centrifuge tube, which 1 ml of ultrapure water was added to the centrifuge tube, mixed with a vortex mixer and completely dissolved, and filtered with a 0.22 μm filter to obtain 20 mg/ml of medicinal liquid spare. The sterile environment of hemoglobin is stabilized by the glycosylation reaction HbCO, pasteurized at 60°C for 12 h to inactivate various viruses and enzymes, and concentrated by ultrafiltration to 42 g/dl. Then the solution was destructurized by adding equimolar 5-pyridoxal phosphate, 1,2-dipalmitoyl-sn-glycero-3-phosphatidylcholine, cholesterol, 1,5-O-dihexadecyl-N-succinyl-L-glutamate (Nippon Fine Chemical Co. Ltd., Osaka, Japan) and 1,2-distearoyl-sn-glycerol-3-phosphatidylethanolamine-N-PEG5000 (NOF Corp., Tokyo, Japan). The concentration was reduced to 10 g/dl by salting out and retained anaerobic. The final HbV physicochemical parameter is P50 (normal oxygen partial pressure is 26.5 mmHg at 50% hemoglobin oxygen saturation) of 17–23 mmHg, in which the diameter of the oxygenated hemoglobin liposome particles is 251 ± 81 nm and the methemoglobin content is less than 10% (Figure 2).

### Infusion of HBOC

The occipital part of the rat was cut longitudinally and the first cervical vertebra of the rat was separated under anesthesia. The burning needle was passed through the first cervical vertebrae of the rat to burn the bilateral vertebral arteries. The mouse was incised in the middle of the neck, and the right common carotid artery and internal and external carotid veins were separated. After that, the PE 10 tube was passed through the external carotid artery to the internal carotid artery. The catheter was indwelled about 19–20 mm. The ischemia + perfusion groups had microcatheters inserted distally six hours after surgery, and were perfused with HBOC. The I + LP group was perfused at a rate of 1.2 ml/h and the I + HP group was perfused at a rate of 4 ml/h. The CTRL group was successfully treated without cerebral infarction.

### Histological observation

Bilateral brain tissue of the infarcted area was removed, sectioned, and examined for pathological differences. Then, TTC staining of the cerebral infarction was performed. Briefly, whole brain tissue was frozen at −20°C for 20 min, then brain tissue removed from the cerebellum was cut into 2 mm thick slices with a razor blade and placed into 12-well plates. 1 ml of 2% TTC dye solution (solebao Cat#3005) was added to each well. The plates were placed in a shaker at 37°C for 30 min in the dark. After the sections were stained, PBS (P1010, Solarblo) was added and removed to wash the tissue. Paraformaldehyde was added to fix, followed by photo recording.

### Western blot analysis

Total protein was isolated, in the manner stated below, from the ischemic core of rat brains. Protein levels of Caspase-3, HIF-1α, and Cytochrome C were obtained. Protease inhibitors (CW2200S, Kangwei Century) were added as per company specifications. After adding the tissue protein at a ratio of 1 : 10 (g/ml) to the protein extraction reagent, the sample was sonicated, centrifuged (10,000 rpm, 20 min)and the supernatant was collected. BCA protein quantification (CW0014, Kangwei Century) at 562 nm was measured with a microplate reader (ZS-2, Beijing Xinfan Electric). An equal amount of protein, for each condition was loaded into different wells of an SDS-PAGE gel and electrophoresed (Table 2). Membranes were then transferred (BIO-RAD No:164-5050, Mini-PROTEAN Tetra Electrophoresis System, Mini Trans-Blot). Then, the one-step fast WB kit was used (Rabbit CW2029, Kangwei Century Mouse CW2030). Briefly, membranes were incubated with antibodies (Table 3), followed by ECL chemiluminescence, and exposure development (CW0049C, Kangwei Century).

**Table 2:** SDS-PAGE separation gel preparation.

Separating glue concentration	Total volume	Required volume of each component (mL)				
		Pure water	30%Acr-bis(29:1)	Separation gel buffer(4×)	APS(10%)	TEMED
10%	5 ml	2.08	1.67	1.25	0.05	0.002
12%	5 ml	1.75	2	1.25	0.05	0.002

**Table 3:** The condition of the target protein.

Target protein	weight(kD)	ratio	Secondary Antibodies	Ratio of secondary antibodies	Incubation conditions
Caspase-3	34	1:500	HRP rabbit	1:200	Incubate overnight at 4°C
Cytochrome C	14	1:5000	HRP rabbit	1:200	Incubate overnight at 4°C
HIF-1α	92	1:1000	HRP rabbit	1:200	Incubate overnight at 4°C

### Real time-PCR

HIF-1α, Bcl-2, and BAX mRNAs were detected by quantitative PCR (Table 4). Fresh brain tissue, 30–50 mg, was added to 1 ml of TRIzol Reagent (5 × 10^6^–1 × 10^7^ cells were combined with 1 ml TRIzol Reagent) and homogenized with a glass homogenizer (XH-B, Jiangsu Kangjian Medical Products Co., Ltd.). The homogenized tissues were left at room temperature for five minutes to completely separate protein and nucleic acid complexes. After centrifugation (12,000 rpm, 10 min), an equal volume of 70% ethanol that was prepared with RNase-free water (Axygen, American) was added to the resulting aqueous solution, and vortexed. Then, samples were added to spin columns that were loaded into collection tubes and centrifuged (12,000 rpm, 20 s). The waste liquid in the collection tubes was discarded after centrifugation. The columns were left at room temperature for 4 min and dried thoroughly. The RNA solutions were centrifuged (12,000rpm, 1 min) and stored at −80°C.

**Table 4:** Primers and base sequences used in these assays.

Names	Forward primers	Reverse primers
β-Actin	gaagatcaagatcattgctcct	tactcctgcttgctgatcca
Bax	agacacctgagctgaccttggag	gttgaagttgccatcagcaaaca
Bcl-2	tgaaccggcatctgcacac	cgtcttcagagacagccaggag
HIF-1α	acagttacaggattccagcagac	gattcatcagtggtggcagttg

### Immunohistochemistry

For immunohistochemical staining, brain tissue from ischemic core was dehydrated, trimmed, embedded, sliced, stained, sealed, and microscopically examined (primary antibody of target gene: Mouse Anti-Caspase-3 antibody/Mouse Anti-Bax antibody, 1 : 50/1 : 100, bsm-33284M/bs-0127M, Boaosen.) (Biotin secondary antibody: Ltd, SP-9001, Beijing Zhongshan Jinqiao Biological Co.). Each section was observed at 100×, and then three fields were selected to observe at 400× with a digital trinocular camera microscope (BA400Digital, McAudi Industrial Group Co., Ltd.). Image-Pro Plus 6.0 image analysis system (Media Cybernetics, United States) was used to measure integrated optical density (IOD) and area of all images collected. The mean density (MD) of each image was calculated by the software. The IOD of three different images was used to calculate an average IOD. The calculated average IOD of each sample was then compared.

### HE and TTC detection

All cases of the ischemic core of rat brain tissue sections were made according to the pathological test SOP program (Dehydration, trimming, embedding, sectioning, staining, mounting, etc.). Each section was observed at 40× before selection of specific areas to take 100× images of lesions.

### Apoptosis detection

Slices of brain tissue were soaked in APES and placed in a 60°C oven for 60 min. After the sections were routinely deparaffinized with water, they were treated with Proteinase K (1245680100, Merck Millipore, United States) added at 37°C for 25 min. After the sections were rinsed with PBS (P1010, Solarblo), 50 μl of TUNEL reaction mixture was added. After the sections were rinsed again, 50 μl solution of converter-POD (10279600, Roche Group) was added. After tissue sections were stained with 50–100 μl DAB (K135925C, Beijing Zhongshan Jinqiao Biological Co., Ltd.), all tissues were observed at 40×. Three regions were randomly selected and imaged at 400×. The number/percentage of apoptotic cells was counted. Finally, the number of apoptotic cells of three regions were averaged.

### TB staining

Brain tissue was soaked with APES, and then placed in an oven at 60°C for 60 min (Leica-2016, Germany). Slices were dewaxed with water, placed in a 1% toluidine blue aqueous (180118, Shanghai Ruji Biotechnology Development Co., Ltd.) solution preheated to 50°C, and warmed to 56°C. Slices were stained for 20 min, and were then washed several times with distilled water. Those slices were soaked in 70% alcohol for 1 min, rapidly dehydrated of anhydrous alcohol, placed in xylene, and sealed by neutral gum. Each section was observed at 40× target lesions. Lesions were observed at 400× an images were taken (BA400Digital, McAudi Industrial Group Co., Ltd.).

### ELISA

The kit (TNF-α: ERC102a Simbrex, IL-6: ERC003 Simbrex, IL-1β: E-EL-R0012c Elirit) was placed at room temperature (20–25°C) for 30 min. Standards or samples were added to each well with 100 µl each, and incubated at room temperature for 120 min. After the liquid was drained, the plate was washed three times and then 100 µl of biotin antibody working solution was added. After the plate was fully cleaned, 100 µl of enzyme conjugate working solution was added for 30 min. After the plate was fully cleaned again, 90 µl of substrate solution was added for 15 min. 50 µl stop solution was added to the kit, and the OD value was immediately measured at 450 nm wavelength.

### Statistics

GraphPadPrism (V7, La Jolla, CA, United States) was used to analyze differences between two groups. Data was expressed as mean ± standard deviation (±SD). A one-way ANOVA was used to compare differences between many groups. A Tukey *post hoc* test was performed if significance was reached in the ANOVA. Nonparametric data were analyzed using Kruskal-Wallis and Dunn's *post hoc* tests. The log-rank comparisons of the groups were used to calculate *p* values. The level of significance was chosen to be at *p* < 0.05.

## Results

### Postoperative neurological score

**Figure 3:**
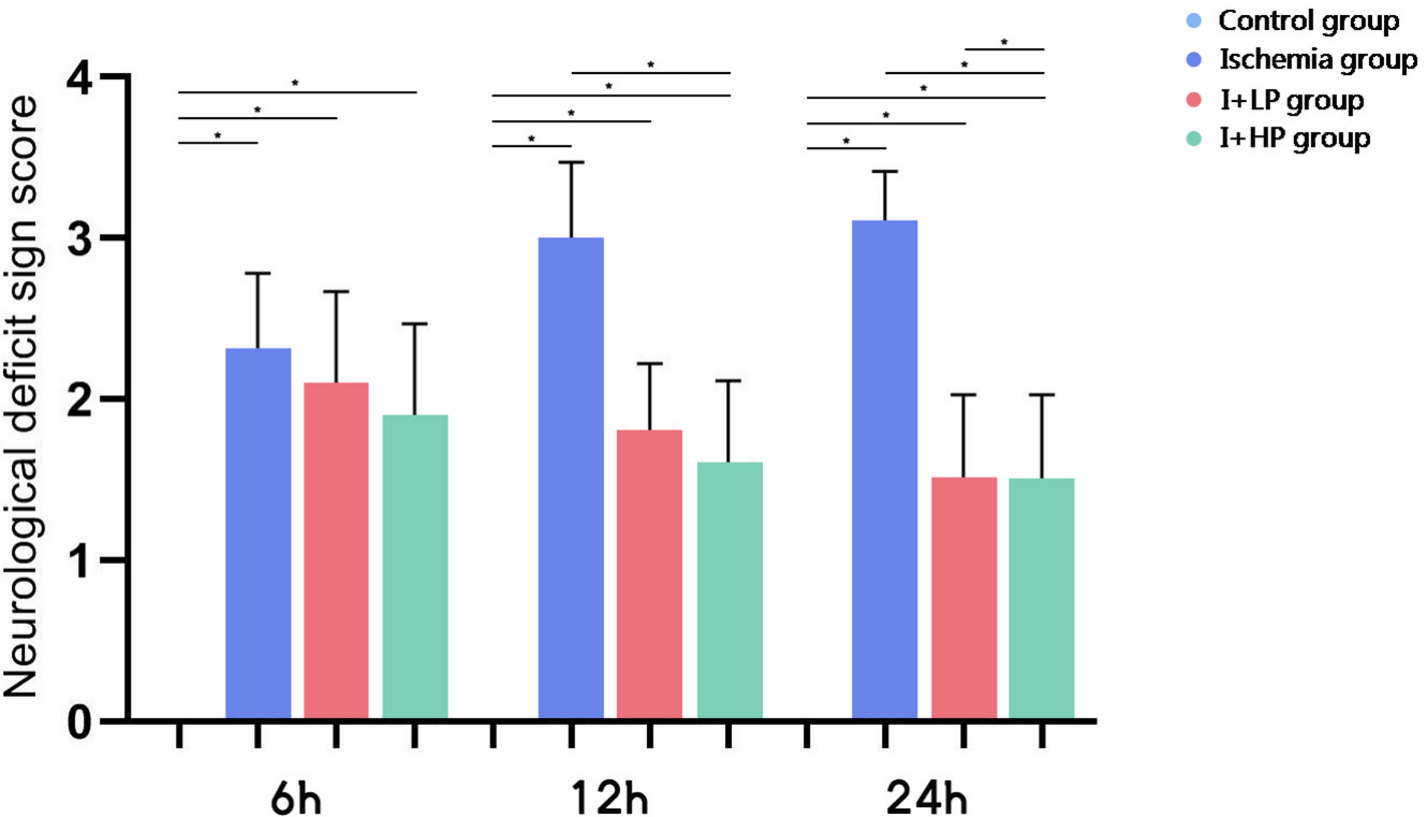
The neurological deficit sign scores of the CTRL group, the I group, the I + LP group and the I + HP group (note: *p < 0.05).

Compared with the CTRL group, the neurological deficit sign scores of the I group, the I + LP group, and the I + HP group were significantly different from each other (*p* < 0.05). Compared with that of the I 6 h group, the neurological deficit sign scores of the I + LP 6 h group and the I + HP 6 h group were not significantly different (*p* > 0.05). Compared with the I 12 h group, the neurological deficit sign scores of the I + HP 12 h group were statistically significant (*p* < 0.05). Compared with that of the I + LP 12 h group, the neurological deficit sign scores of the I + HP 12 h group were not significantly different (*p* > 0.05). Compared with the I 24 h group, the neurological deficit sign scores of the I + LP 24 h group and the I + HP 24 h group were statistically significant (*p* < 0.05). Compared with those of the I 24 h group and the I + LP 24 h group, the neurological deficit sign scores of the I + HP 24 h group were significantly different (*p* < 0.05) (Figure 3).

### HBOC perfusion decreases apoptotic proteins, apoptotic mRNA, inflammatory cytokines, and apoptotic index after ischemia

**Figure 4:**
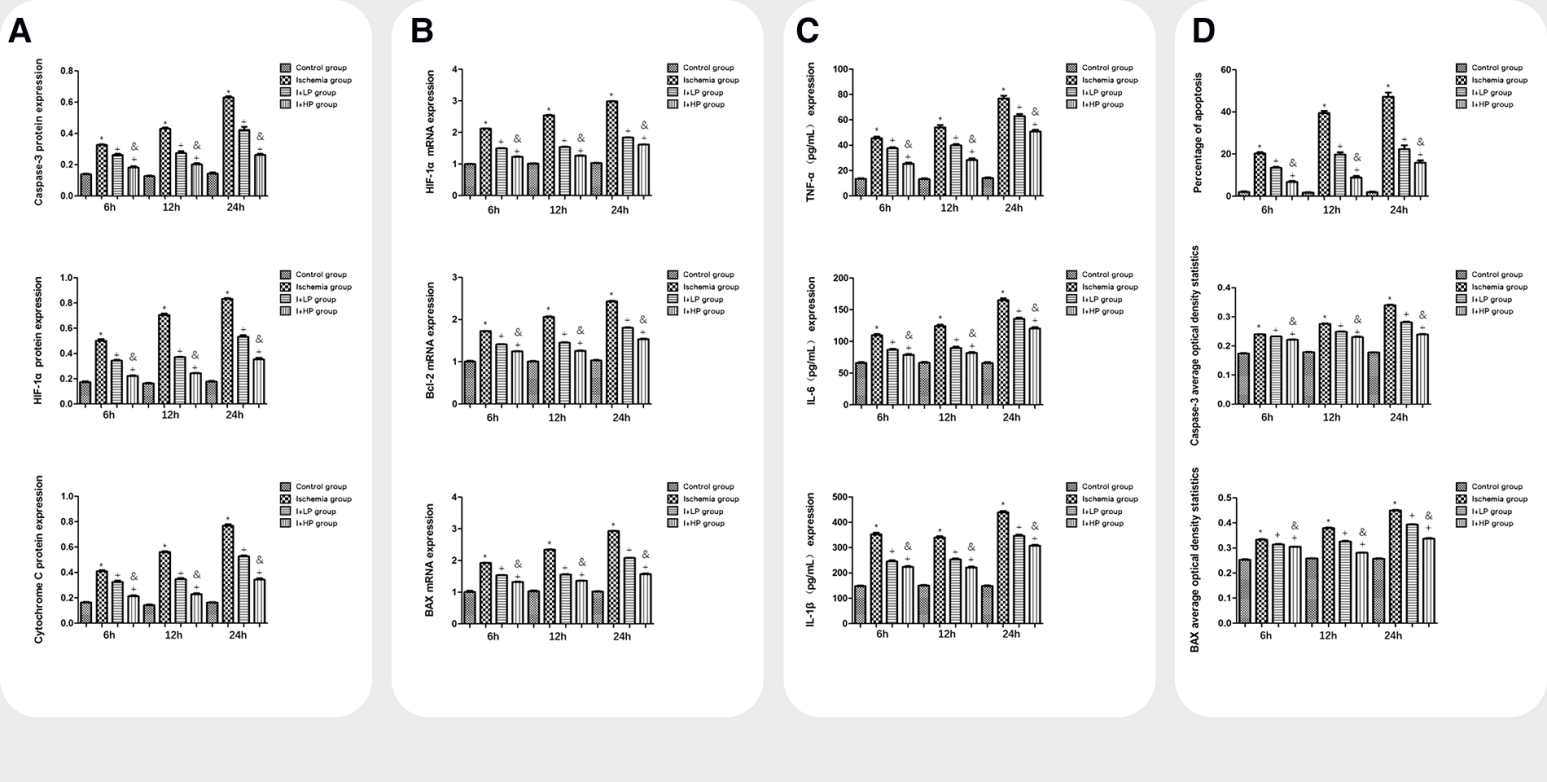
A comparisons in expression of caspase-3, HIF-1α, and cytochrome C protein in brain tissues of each group. B Comparisons in expression of HIF-1α, Bcl-2, and BAX mRNA in brain tissues of each group. C Comparisons in TNF-α, IL-6, and IL-1β expression in the serum of each group. D Comparisons in the percentage of apoptotic cells, Caspase-3, and BAX average optical density in brain tissue of rats in each group (Note: *compared with the control, *p* < 0.05; *compared with the ischemia, *p* < 0.05; and compared with low perfusion group, *p* < 0.05).

**Figure 5:**
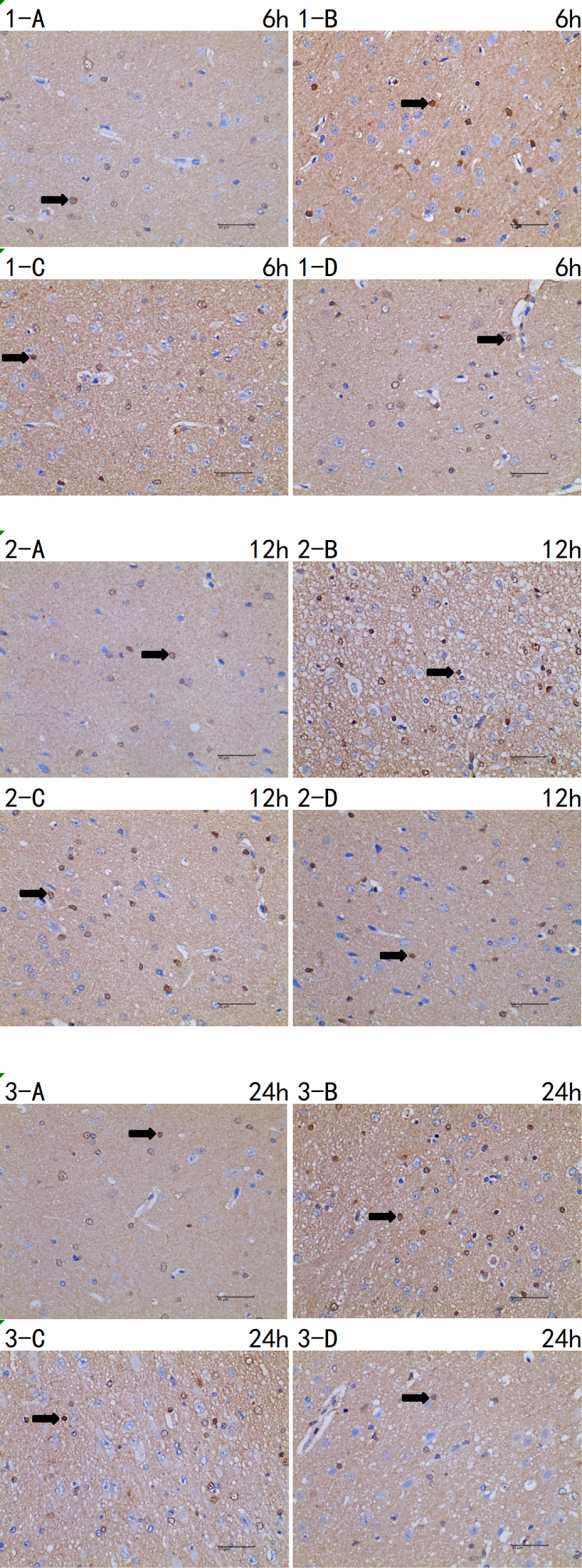
Immunohistochemistry of caspase-3 6 h/12 h/24 h × 400 test report (A CTRL group, B I group, C I + LP group, D I + HP group) positive cells (**→**) are yellow or brown, and caspase-3 positive products are mainly distributed in cytoplasm and intercellular substance.

**Figure 6:**
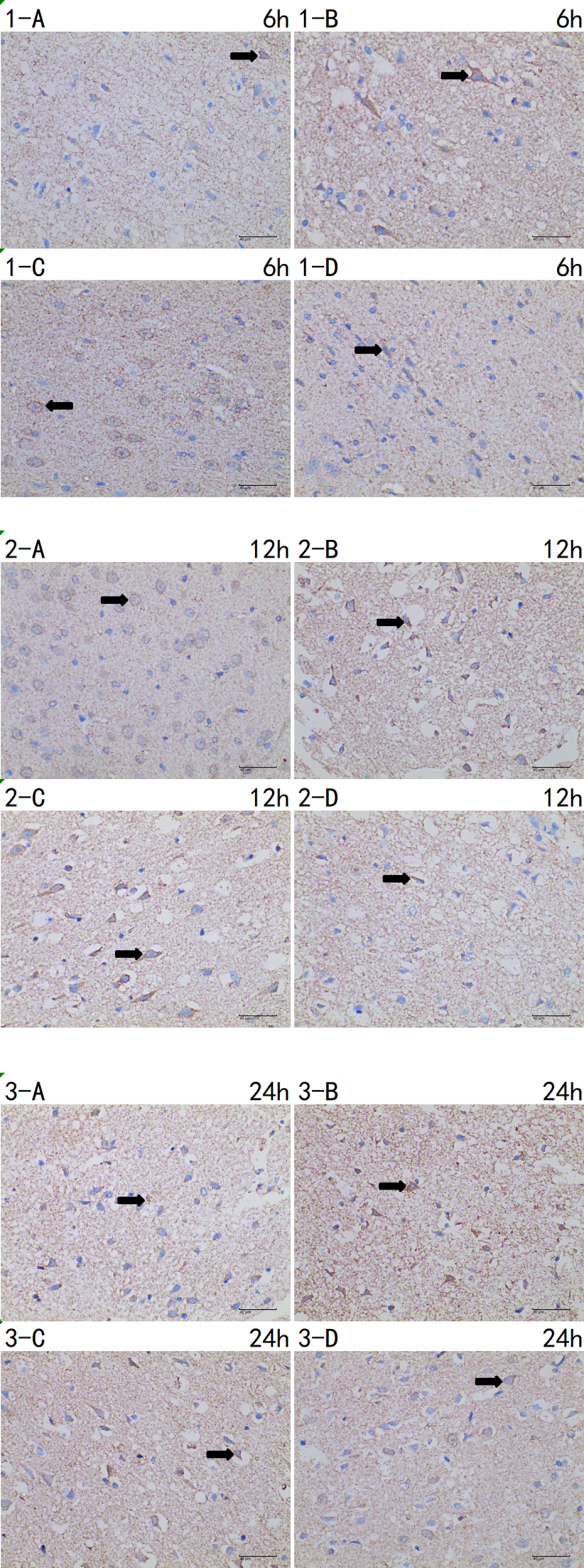
Immunohistochemistry of BAX 6 h/12 h/24 h × 400 test report (A CTRL group, B I group, C I + LP group, D I + HP group). Positive cells (**→**) are yellow or brown, and BAX positive products are mainly distributed in cytoplasm and intercellular substance.

**Figure 7:**
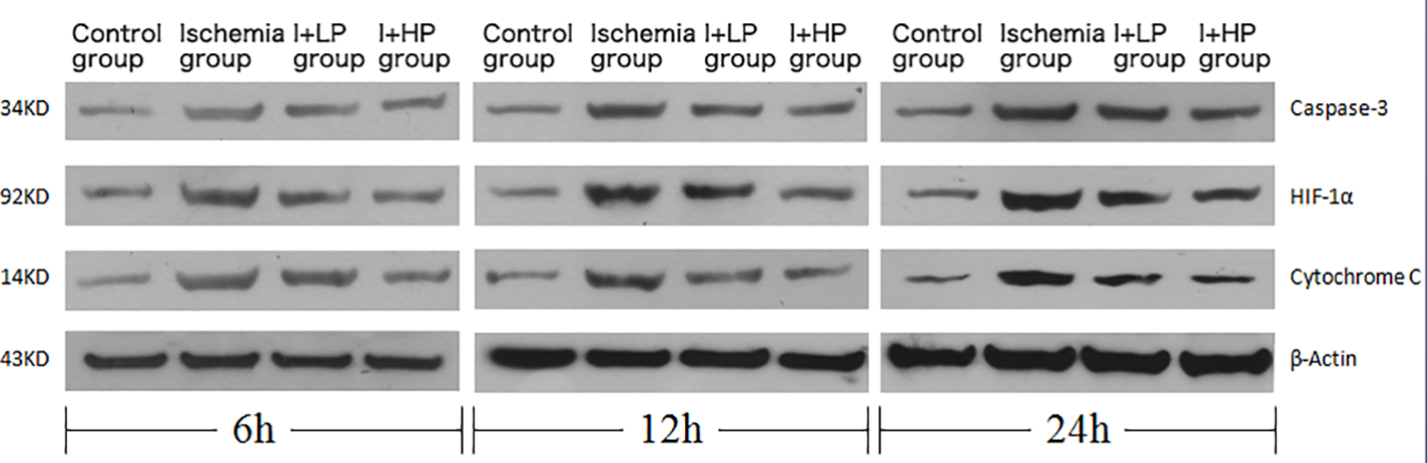
Changes in expression of caspase-3, HIF-1α, cytochrome C protein in brain tissues of each group.

Compared with the I and the I + LP group, apoptotic proteins (Caspase-3, HIF-1α, and Cytochrome C) in the I + HP group were significantly decreased (Figure 4A). HIF-1α, Bcl-2, and BAX mRNA were significantly lower in the same group (Figure 4B). ELISA revealed lower levels of TNF-α, IL-6, and IL-1β in the I + HP group (Figure 4C). Caspase-3 average optical density, BAX average optical density, and apoptotic index decreased in the I + HP group as well (Figure 4D). Immunohistochemistry of Caspase-3 and BAX showed that perfusion of HBOC decreased the number of positive cells (Figures 5, 6). The expression of Caspase-3, HIF-1α, Cytochrome C protein was inversely proportional to the dose of HBOC on the western blot (Figure 7).

### HBOC perfusion decreases hypoxia-induced apoptosis in brain cells

**Figure 8:**
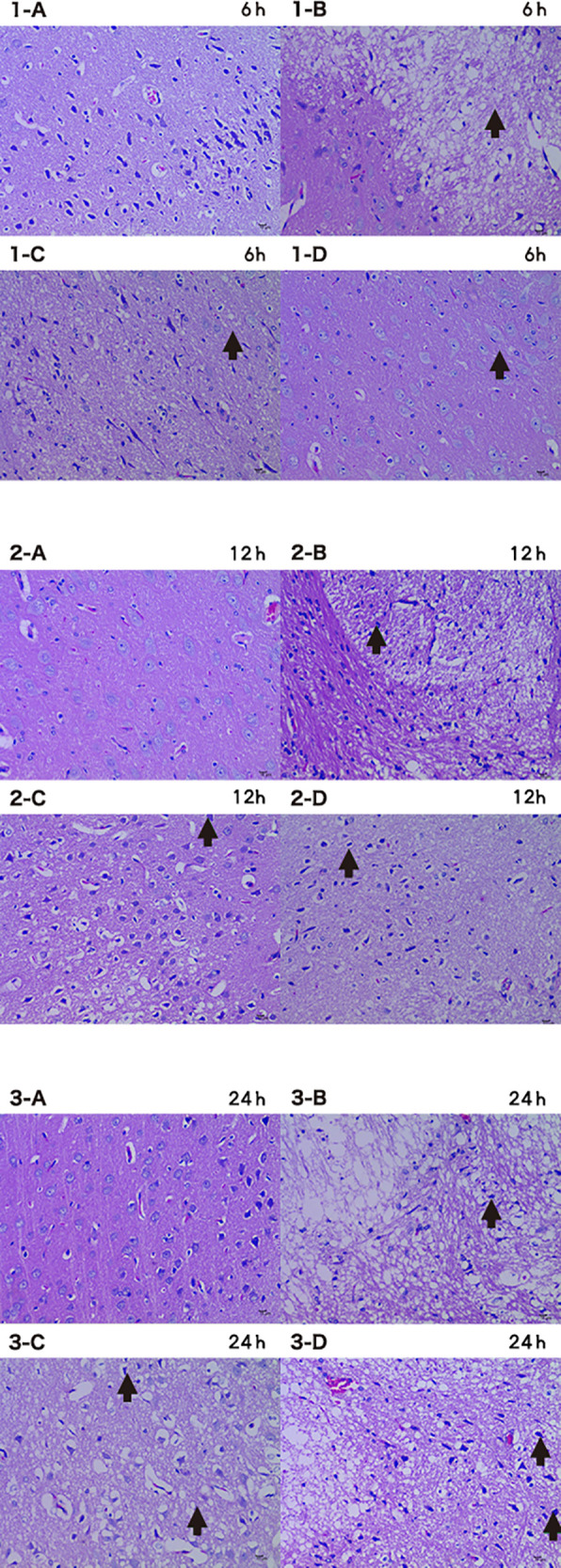
Brain tissue HE 6 h/12 h/24 h × 200 (A CTRL group, B I group, C I + LP group, D I + HP group) neurons showed damage with missing, vacuolated, and necrotic cells (**→**).

The I group had a large number of neurons which were missing, necrotic, and vacuolated in the basal ganglia (Figure 8). The neurons in the basal ganglia of the I + HP 24 h group had less necrosis and cerebral infarction as compared to the CTRL group (Figure 8). The brain white matter area neurons of the I + LP 24 h group showed a large degree of damage with missing, vacuolated, and necrotic cells while the I + HP group did not (Figure 8).

### HBOC perfusion delays brain damage due to ischemia

**Figure 9:**
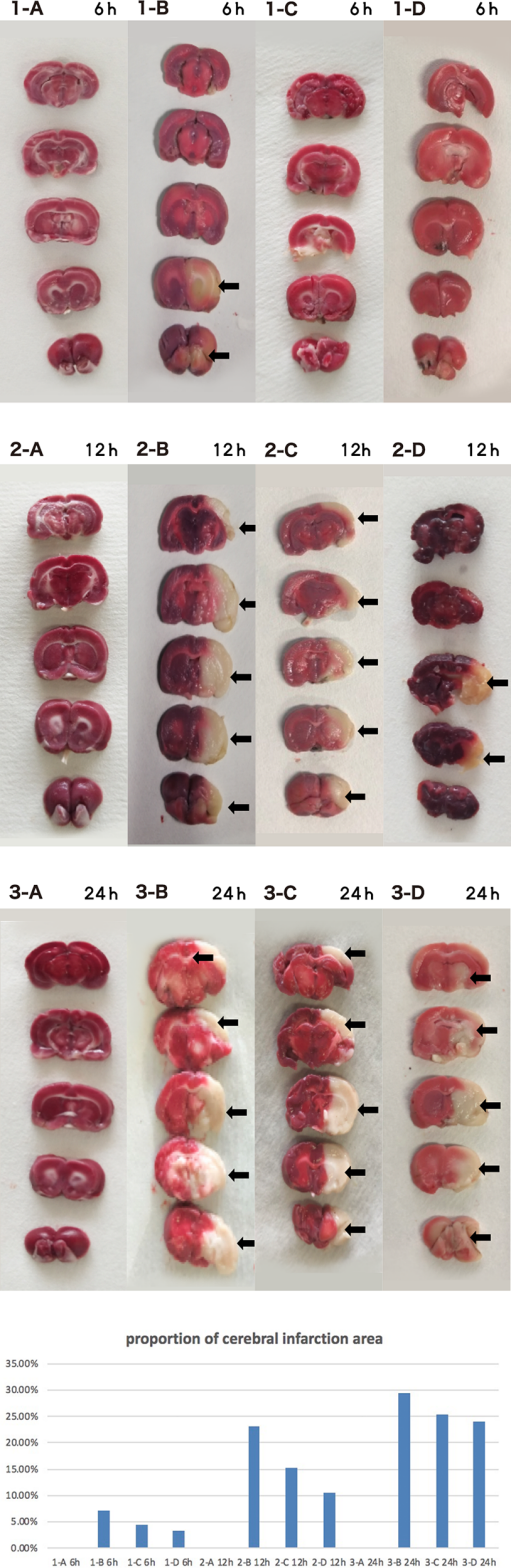
Brain tissue TTC 6 h/12 h/24 h (A CTRL group, B I group, C I + LP group, D I + HP group) the cerebral infarct area (**→**) is the white area.

Control rat brains showed no obvious injury. The infarct size of the I group gradually increased with time, as expected. The I + LP group had less increase with time than the I group but yet still more than the high-perfusion group, indicating higher profusion for a longer time is better (Figure 9).

### HBOC perfusion saves brain from ischemic damage

**Figure 10:**
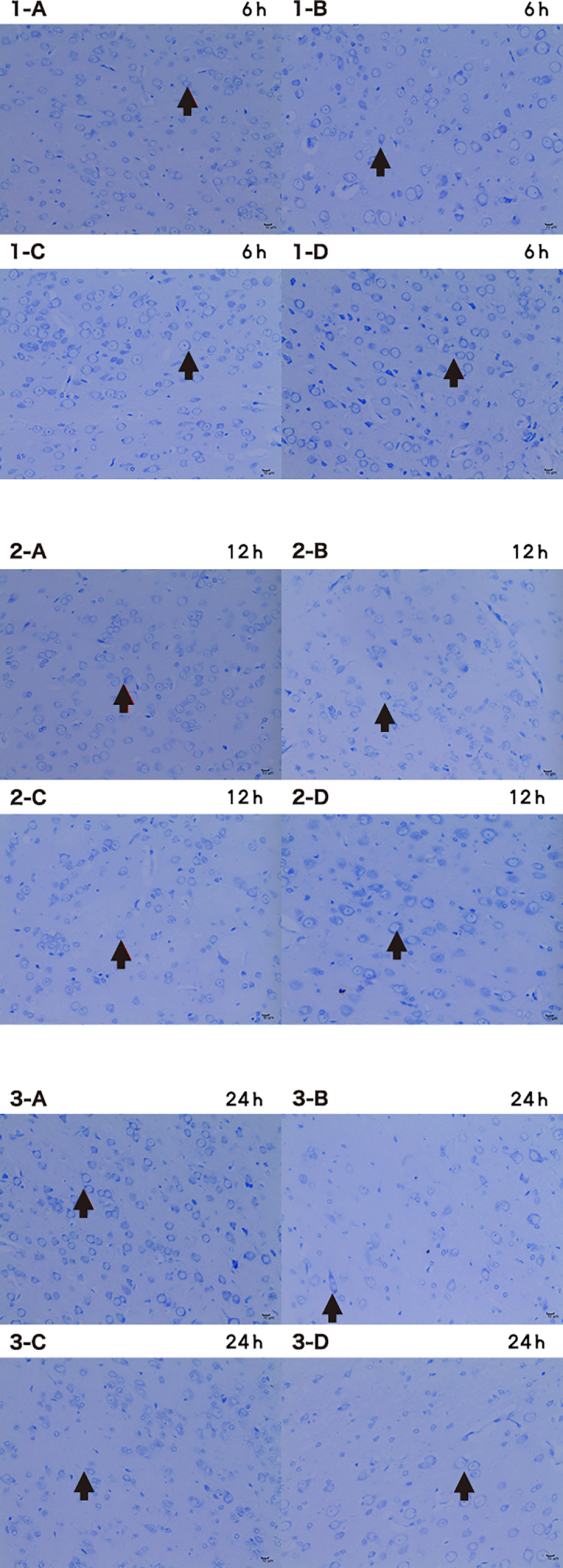
Microscopic image of nissl stained cells 6 h/12 h/24 h × 200 (A CTRL group, B I group, C I + LP group, D I + HP group) the nucleus of nissl bodies (**→**) is a dark blue round or oval cell, and the cytoplasm is full of thick purple-red metachromatic particles, which are mainly distributed in the interstitium. The Nissl bodies are dark blue with a colorless background.

Compared with control, the number of Nissl-stained cells was significantly decreased at 6, 12, and 24 h of perfusion (*p* < 0.05) (Figure 10).

## Discussion

**Figure 11:**
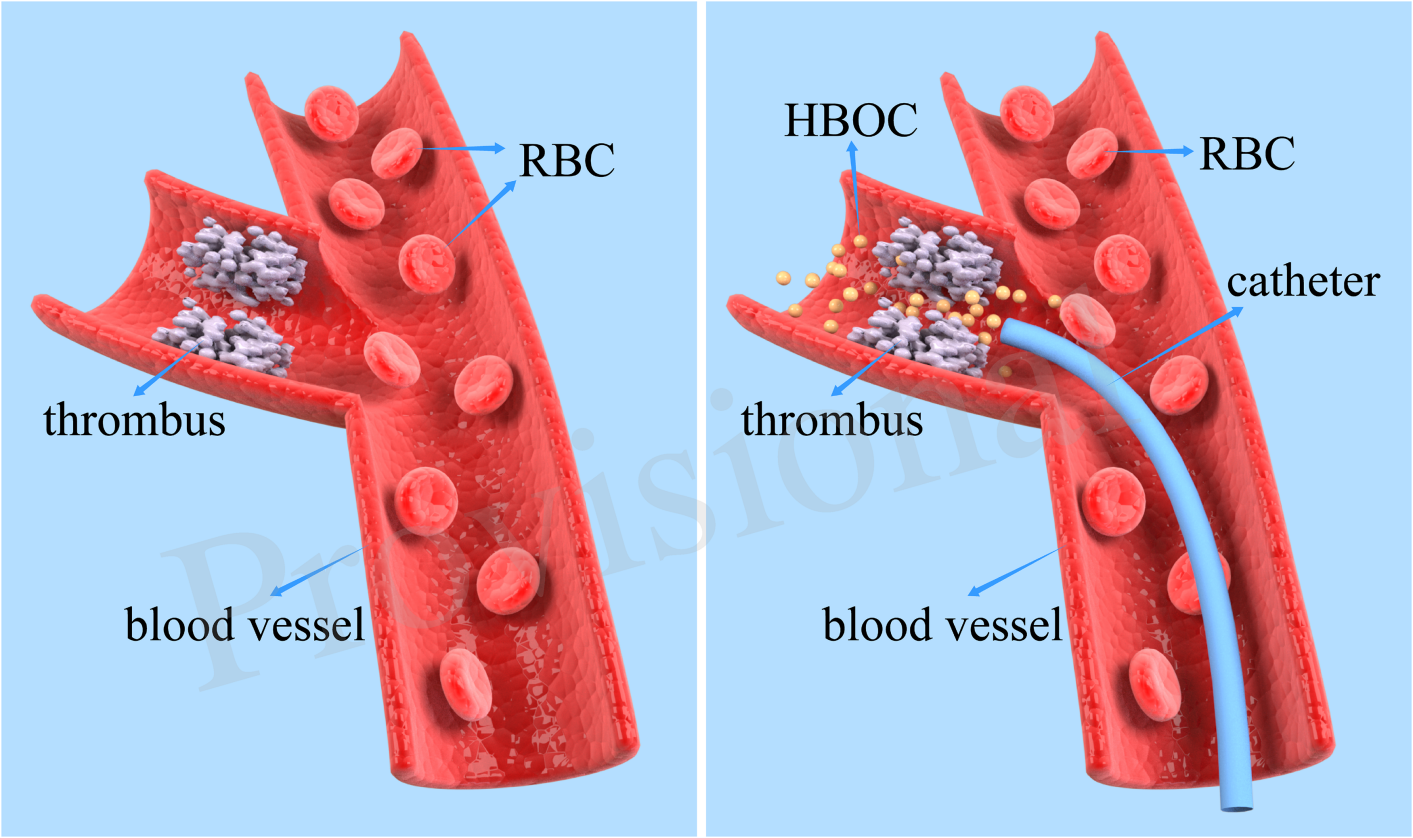
The pathway of neurointerventional infusion of HBOC.

In this study, a neurological intervention technique was used to send HBOC to cerebral infarction sites *via* a microcatheter, in order to protect ischemic brain tissue (Figure 11). When there is no thrombolysis and no thrombus removal, a variety of indicators and detection methods are used to directly and indirectly observe whether a method can effectively alleviate the symptoms of cerebral ischemia, thereby alleviating the death of cerebral nerve cells caused by hypoxia.

In rats with cerebral infarction, the expression of Caspase-3 and Cytochrome C were significantly increased, indicating apoptotic conditions were present. With the increase of HBOC perfusion time, the expression of Caspase-3 and Cytochrome C decreased, indicating that cerebral ischemia and hypoxia were relieved and subsequent apoptosis prevented. The expression of HIF-1α subunit was virtually undetectable in cells under normal oxygen saturation. Degradation of the HIF-1α subunit is inhibited in the hypoxic state, leading to subunit dimerization and nuclear translocation, regulating the transcription of multiple genes. Increased expression of HIF-1α has neuroprotective effects on brain tissue (5). This study indirectly suggests that ischemic brain tissue was receiving oxygen through perfusion.

Inflammatory factors TNF-α, IL-6, and IL-1β participate in the process of early inflammatory injury of cerebral infarction. With a significant increase, they are sensitive biochemical indicators of early infarction. This study shows that administration of HBOC can help prevent the damaging effects of ischemia by reducing these inflammatory cytokines. Since Bcl-2 can inhibit cell death caused by a variety of cytotoxic factors, its overexpression gives cells resistance to most cytotoxins. The increased amount of Bcl-2 in HBOC is beneficial in this respect to further defend the brain tissue against stroke (9). Interestingly, this study found that BAX mRNA expression increased after hypoxia, and cell apoptosis reduced. Studies that have evaluated the efficacy of HBOC transfusion doses through the activation of intrinsic anti-apoptotic mechanisms and their role in reducing tissue damage are in accordance with ours. The decline in these indicators after HBOC transfusion represents an effective treatment. The neurological function of rats with cerebral infarction has also been improved after treatment. Therefore, appropriate dose administration is beneficial to delay the process of ischemic stroke. The range of dose were designed to be much less than normal cerebral blood flow in mice in this study due to safety. The optimal dose for mice and the appropriate dose for human cerebral blood vessels need to be further studied in the future.

HBOC can not only be stored at room temperature for a long time, but also does not need to be cross-matched before entering the human body because HBOC does not induce the immune system to produce antibodies, so it is considered to be safe and effective in acute cerebral infarction events (10). The HBOC has small volume and can pass through tiny blood vessels that ordinary blood cells cannot pass, and cross the blood-brain barrier to reduce ischemic reperfusion injury (5). However, little is known about the time window of HBOC treatment in patients with cerebral ischemia. HSIA and MA found in animal models of traumatic brain injury that HBOC may have the effect of protecting cerebrovascular, which may be the reason of reduced infarcted brain tissue (11). KAKEHATA et al. showed that HBOC can improve hippocampal dysfunction after acute ischemic stroke in rats, and HBOC may have the potential to protect the brain and improve neurological complications related to cerebral infarction (12). ZHANG et al. found that infusion of HBOC in a rat model of middle cerebral artery occlusion could reduce infarct size, maintain cerebral vasodilation and improve collateral perfusion (13). KAWAGUCHI et al. conducted experiments on non-human primates and found that HBOC still has a role in early brain protection after ischemia in monkeys (4, 6). In this study, a rat model was used to study the relationship between infusion time/rate and efficacy in preventing injury in cerebral ischemia. Many results including HE staining, TTC staining, and Nissl staining have proven the efficacy of HBOC supply to ischemic brain tissue. This study shows that although cerebral blood flow is reduced after an ischemic attack, HBOC can still relieve cerebral infarction with interventional mirocatheter technology.

Previous studies have used blood products to treat cerebral infarction, but the researchs of interventional technology infusion of HBOC for acute cerebral infarction are very rare (14, 15). HBOC can not only increase the oxygen-carrying function of blood, but also its ability to release oxygen to tissues is stronger than that of hemoglobin in red blood cells (9). It is speculated that the time window of application of HBOC may be longer than that of tissue plasminogen activator (tPA) and there were no obvious complications through this experiment. In the early stage of a thrombotic event, HBOC in the systemic circulation can reach the ischemic brain area through the infarct site where red blood cells cannot pass and reduce ischemia-hypoxic injury due to its small molecular weight. It has been confirmed in animal experiments that lower doses of HBOC (10 ml/kg) can significantly reduce the scope of cerebral infarction in experimental animal models of cerebral ischemia and play a significant role in brain protection (16, 17). In this study, the use of microcatheter technology to accurately deliver HBOC to ischemic brain tissue makes the treatment more facile and effective than previous, ordinary treatments. The disadvantage of this method is that the oxygen content of HBOC cannot be estimated, which has a certain degree of impact on other tests. The fact that basic blood pressure conditions of patients were different and that blood pressure changes after HBOC is taken, may have had an impact on this study. Blood pressure levels, which may affect blood flow in blood vessels to perfuse the ischemic brain tissue, was not effectively controlled in this study. Especially in the case of prolonged vascular infarction, dose and time of HBOC transfusion still need to be elucidated. These experiments show faster rates of HBOC infusion save more cerebral infarcted tissue. It is considered that vasospasm causes this phenomenon since the area of damage was very non-uniform in Figure 6B. Another limitation of the study is that it focused only on neuronal changes over a short time period, so it cannot prove the long-term effect of HBOC in the treatment of acute cerebral infarction. HBOC as a blood substitute may cause kidney damage. A longer time period and larger sample size need to be evaluated in future studies.This experiment was applied to SD rats, which have life functions that are different than humans. These experiments provide a direction for future work, but clinical application of this technology to humans still needs to be explored. Once the technology is applied in clinical work, the diagnosis and treatment of acute cerebral infarction will more than likely have a decisive positive change.

In this study, a variety of indicators were used to verify methods of treating cerebral infarction with HBOC transfusion, but the prognosis and recovery of neural functions were not analyzed (17). Despite the above shortcomings, the use of interventional microcatheter technology to perfuse HBOC for treatment of cerebral infarctions has certain advantages and possibilities (18–20). If the same blood type is infused into human cerebral infarction by catheter in the future, this method will have great prospects. Further research is needed employ this method more effectively in the clinic.

## Conclusion

Acute cerebral infarction in rats can be delayed by perfusing HBOC through a microcatheter in a time- and rate-controlled manner. Brain structure preservation through the lowering of inflammation and apoptosis after induced-infarction was observed in rats. Further research is needed to make this treatment available to patients.

## Data Availability

'The original contributions presented in the study are included in the article/Supplementary Materials, further inquiries can be directed to the corresponding author.
